# Editorial: Childhood Cancer in sub-Saharan Africa

**DOI:** 10.3332/ecancer.2017.ed69

**Published:** 2017-07-28

**Authors:** Donald Maxwell Parkin, Cristina Stefan

**Affiliations:** 1 African Cancer Registry Network, INCTR, Prama House, 267 Banbury Road, Oxford OX2 7HT, United Kingdom; 2 CTSU, University of Oxford, Oxford OX3 7LF, United Kingdom; 3 Medical Research Council, PO Box 19070, Tygerberg 7505, Republic of South Africa

**Keywords:** childhood, cancer, sub-Saharan Africa, registry, incidence

## Abstract

Measurement of incidence rates of childhood cancer in Africa is difficult. The study ‘Cancer of Childhood in sub Saharan Africa’ [Stefan C, Bray F, Ferlay J, Parkin DM and Liu B (2017) **Cancer of Childhood in sub-Saharan Africa** *ecancer* **11**(755)] brings together results from 16 population-based registries which, as members of the African Cancer Registry Network (AFCRN), have been evaluated as achieving adequate coverage of their target population. The cancers are classified according to the third revision of the International Classification of Childhood Cancer (ICCC-3) and recorded rates in Africa are compared with those in childhood populations in the UK, France, and the USA.

It is clear that, in many centres, lack of adequate diagnostic and treatment facilities, leads to under-diagnosis (and enumeration) of leukaemias and brain cancers. However, for several childhood cancers, incidence rates in Africa are higher than those in high income countries. This applies to infection-related cancers such as Kaposi sarcoma, Burkitt lymphoma, Hodgkin lymphoma and hepatocellular carcinoma, and also to two common embryonal cancers—retinoblastoma and nephroblastoma. These (and other) observations are unlikely to be artefact, and are of considerable interest when considering possible aetiological factors, including ethnic differences in risk (and hence genetic/familial antecedents).

The data reported are the most extensive so far available on the incidence of cancer in sub Saharan Africa, and clearly indicate the need for more resources to be devoted to cancer registration, especially in the childhood age range, as part of an overall programme to improve the availability of diagnosis and treatment of this group of cancers, many of which have—potentially—an excellent prognosis.

There are few clear environmental exposures influencing the probability of developing cancer in the early years of life; the great majority of malignancies must surely be due to random chance (of one or several mutational events), although pre-existing genetic profiles influence the probability of such events. Geographic variations in incidence may therefore be informative not only about localised exposures (in environment or lifestyle), but also about the spatial distribution of relevant genetic profiles. There is more genetic diversity between populations living within the African continent than between Africans and the rest of mankind [3], so that Africa may be a fruitful place to look for differences in the risk (incidence) of childhood cancer.

Cancer incidence can only be obtained via population-based cancer registration, which requires meticulous collection of data on ALL new cases arising in a given population of known size and composition (by age and sex, at least). Some clues about the relative frequency of different cancers can be gleaned from clinical (or pathology) series, but these are inevitably biased because the population from which the cases come cannot be defined (numerically), and the selective factors resulting in referral to specialist care.

Studies of the incidence of cancer in childhood in sub-Saharan Africa are even more difficult than those of adults. Although childhood cancers are relatively more common in sub-Saharan Africa than in high income countries (4.6% of cancers occur at ages 0–14, compared with 0.5% in high income countries), they are still a relatively uncommon cause of morbidity—on average, only 33 cases will occur each year in a population of one million. In consequence, population-based studies must involve rather large populations (or long time periods) so that sufficient cases can be assembled to permit calculation of valid rates.

In the paper ‘Cancer of Childhood in sub Saharan Africa’ [1], results are presented from population-based registries which, as members of the African Cancer Registry Network (AFCRN), have been evaluated as achieving at least 70% coverage of their target population (that is to say, identifying 70% of the actual incident cancers overall). 15 centres provided a minimum of 150 paediatric cancer cases, in a recent period of no longer that 10 years duration^1^. The recent publication ‘International incidence of childhood cancer, 2001–2010’ [22] included pooled data from 6 of these registries, with 70% of the cases derived from one registry (the South African Children’s Cancer Registry).

The cancers have been classified according to the third revision of the International Classification of Childhood Cancer (ICCC-3) [21]. There are 12 main diagnostic groups, as follows: leukaemia; lymphomas and reticuloendothelial neoplasms; central nervous system (CNS) and miscellaneous intracranial and intraspinal neoplasms; sympathetic nervous system tumours; retinoblastoma; renal tumours; hepatic tumours; malignant bone tumours; soft-tissue sarcomas; germ-cell, trophoblastic and other gonadal neoplasms; carcinomas and other malignant epithelial neoplasms; other and unspecified malignant neoplasms. For six of these broad categories, results are shown for a number of subgroups in addition.

It should be noted that, although all of the contributing registries meet an overall criterion of completeness of ascertainment of cancer cases (70% or more), this may not apply to specific types of cancer, or even to cancers of childhood in general. Lack of specialists and diagnostic facilities in low income settings make it notoriously difficult to diagnose some of the more common malignancies of children (notably, leukaemias and brain cancers), while in the face of still high mortality from infectious diseases (and other causes) in infancy, cancers presenting at very young ages may be unrecognised [10, 12]. However, some cancer types appear have a higher incidence in sub Saharan Africa registries than in high income countries, so it is clearly not wise to dismiss all lower incidence rates as due to artefact.

The recorded incidence rates in sub-Saharan Africa are compared with four comparison childhood populations—the UK, France, and the USA (white and black). For these populations—as has been noted previously [15]—the total incidence of childhood cancer varies rather little, with a cumulative rate (0–14) of about 2 per 1000 (rather lower—1.7 per 1000 in US blacks). The rates in the African populations are much more variable. Although many are around 1.5 per 1000, some of the centres (e.g. Gambia, Conakry, Niamey, Eastern Cape) report very low rates, which is generally the consequence of failing to register (or, probably, to diagnose) childhood leukaemias and brain tumours. Conversely, in regions where there in a high risk of certain infection-related cancers—Kaposi sarcoma and Burkitt lymphoma - the overall incidence is very high (4 per 1000 in Blantyre, Malawi, for example). Indeed, it is the huge variability in the incidence of these latter two cancers that is perhaps the most striking result of the comparisons presented.

Prior to the onset of the epidemic of HIV-AIDS, the incidence of KS in childhood in East and Southern Africa was around 30–40 per million (cumulative rate). In ‘Cancer of childhood in Sub-Saharan Africa’, data from registries in the same area for the 2000’s show rates between 170 per million (Eldoret, Kenya) and 441 per million (Kampala, Uganda). Although very high rates of HIV infection are observed in South Africa (and, to a lesser extent, in Western Africa), endemic KS has never been common in these areas [5], and childhood KS remains relatively rare. This may in part relate to lower population prevalence of infection with KSHV [4], although this is probably not the entire explanation—at least, not for adult KS [7].

Burkitt lymphoma in Africa is generally diagnosed by light microscopy, rarely making use of confirmatory immunohistochemistry or cytogenetics. Despite the resultant misclassifications with other types of lymphoma (or even non-malignant lymphocytic proliferations) [14], the geographic distribution of childhood BL corresponds quite closely to that described in the early descriptions of the disease. Wright (1967) [27] for example, characterised this as ‘a zone 15’ north and south of the equator (with a prolongation southward into Mozambique to the east), although, even within this area BL is infrequent in high-altitude regions, such as Rwanda and Burundi, the Kenya Highlands and the plateaux of Zambia and Zimbabwe. The aetiological role of malaria (in addition to Epstein Barr virus) in defining this striking geographic distribution is summarised in the relevant section of ‘Cancer of childhood in sub-Saharan Africa’.

Two other cancers related to infection—Hodgkin lymphoma (HL) and hepatocellular carcinoma show features that differ in African children from those in higher income countries. HL is rather rare in childhood, so that the numbers of cases for study are few, but the results do suggest that it is more common in Africa, particularly in younger children (under age 10), and in boys. This fits with early observations that in low income settings, HL is usually EBV-related, more common in young children, in boys and mainly presenting as mixed cellularity HD [24]. Hepatocellular carcinoma is rare in childhood in Europe and North America, but a relatively high incidence is observed in regions of the world with high rates of adult liver cancer, including sub-Saharan Africa (especially West Africa). Most childhood cases of liver cancer in these areas occur in chronic carriers of hepatitis B [28, 29], and most cases are in older children (10–14 years).

Two embryonal tumors show rates in African children that are rather higher—in most centres—than those in Europe or the USA: retinoblastoma and nephroblastoma, while a third—neuroblastoma, has been considered to be rare. The reasons for the rather elevated rates of retinoblastoma are obscure. It seems likely, based on clinical series, that it is due to an excess of unilateral (rather than bilateral) disease, which might indicate an environmental factor (rather than higher prevalence in African populations of mutations in the retinoblastoma gene). The rather late age at diagnosis in Africa is in part due to this excess of non-heredity disease (which occurs at older ages than bilateral cases), but is undoubtedly also due to late clinical diagnosis. In the past, nephroblastoma (Wilms tumour) was reported to be rather more common in populations of African ancestry [2, 23], although currently in the USA there seems to be no difference between whites and blacks. Results from sub-Saharan African registries are varied, with high rates from those in eastern Africa, moderate rates in southern African, and varying levels in West Africa. Unlike retinoblastoma, few cases can be identified as directly hereditary, and there are really no clear environmental factors influencing risk. The reasons for the ethnic (and possibly, regional) variations in risk, are therefore obscure. As for neuroblastoma, it has long been suspected to be a relatively rare cancer of African children. Miller (1989) [13] reviewed published data and drew attention to the very low frequency (<1% of childhood cancers) in east and central Africa; data from West Africa seemed to indicate similar rarity [16]. The more recent data in this monograph still suggest a low incidence in African children, although there is little difference in incidence between black and white children in the USA [19] or UK [23]. Since most neuroblastomas occur in the first year of life, it is possible that they are under-diagnosed as a cause of morbidity and mortality in Africa, and/or confused with other small cell sarcomas [25], but since almost nothing is known of aetiological factors, the possibility of ethnic differences in risk (and hence genetic/familial antecedents) is clearly of interest.

Globally, the most important cancers of childhood are leukaemias (32.3% of childhood cancers) and tumours of the central nervous system (20.4%) [22], but, as noted, these cancer types are frequently underdiagnosed and underreported in sub-Saharan Africa. With respect to leukaemia, it is quite likely that, in the absence of specialist facilities, many cases remain undiagnosed, and cancer registries may fail to identify those that are. Nevertheless, even taking these factors into account, the results are consistent with other reports which suggest that incidence rates—particularly of acute lymphoblastic leukaemia—are low in African children. In high income countries, the high incidence of common (precursor B cell) acute lymphoblastic leukaemia was not present decades earlier, but seems to be a feature of increasing socio-economic development [6, 17]. Various hypotheses have been proposed to explain this phenomenon [9].

## Conclusions

The data in ‘Childhood cancer in sub-Saharan Africa’ are the most extensive so far available on the incidence of cancer in sub-Saharan Africa. Although the overall incidence rates may be lower than those observed in high-income countries, the large proportion of the population that are children (43%) means that childhood cancer is proportionately more common (4.6% of cancers in sub Saharan Africa v 0.5% in high income countries)—an estimated total of about 29,000 new cases in 2012 [8]. To date, there are almost no population-based data on survival from childhood cancer in sub-Saharan Africa, available statistics are based simply on opinion, or results from selected clinical series, which suggest a rather dismal outcome. This is perhaps unsurprising, given that facilities for paediatric oncology are few in number, and small in size [11, 18]. Although childhood cancers are eminently treatable, the costs are relatively high, alliances between public, private, and international agencies can improve the outcome of children with cancer in these countries (e.g. the ‘My Child Matters’ program sponsored by Sanofi-Espoir and UICC).

## Footnotes

1 Results from one smaller registry (Eastern Cape of South Africa), with 113 cases in 10 years are also presented

## References

[1] Stefan C, Bray F, Ferlay J, Parkin DM, Liu B (2017). Cancer of Childhood in sub-Saharan Africa. ecancer.

[2] Breslow N (1994). Ethnic variation in the incidence, diagnosis, prognosis, and follow-up of children with Wilms’ tumor. J Natl Cancer Inst.

[3] Cavalli-Sofrza LL (1997). Genes, peoples and languages. Proc.Natl Acad Sci USA.

[4] Dollard SC (2010). Substantial regional differences in human herpesvirus 8 seroprevalence in sub-Saharan Africa: insights on the origin of the “Kaposi’s sarcoma belt”. Int J Cancer.

[5] Cook-Mozaffari P (1998). The geographical distribution of Kaposi’s sarcoma and of lymphomas in Africa before the AIDS epidemic. Br J Cancer.

[6] Court Brown WM, Doll R (1961). Leukaemia in childhood and young adult life. BMJ.

[7] Dedicoat M, Newton R (2003). Review of the distribution of Kaposi’s sarcoma-associated herpesvirus (KSHV) in Africa in relation to the incidence of Kaposi’s sarcoma. Br J Cancer.

[8] Ferlay J (2013). GLOBOCAN 2012 v1.0, Cancer Incidence and Mortality Worldwide: IARC CancerBase No. 11.

[9] Greaves MF (1997). Aetiology of acute leukaemia. Lancet.

[10] Howard SC (2008). Childhood cancer epidemiology in low-income countries. Cancer.

[11] Kruger M (2014). Childhood cancer in Africa. Pediatr Blood Cancer.

[12] Magrath I (2013). Paediatric cancer in low-income and middle-income countries. Lancet Oncol.

[13] Miller RW (1989). No neuroblastoma in Zaire. Lancet.

[14] Ogwang MD (2011). Accuracy of Burkitt lymphoma diagnosis in constrained pathology settings: importance to epidemiology. Arch Pathol Lab Med.

[15] Parkin DM (1998). International incidence of childhood cancer, vol II.

[16] Parkin DM (2003). Cancer in Africa: Epidemiology and Prevention.

[17] Ramot B, Magrath I (1982). Hypothesis: the environment is a major determinant of the immunological sub-type of lymphoma and acute lymphoblastic leukaemia in children. Br J Haematol.

[18] Ribeiro RC (2008). Baseline status of paediatric oncology care in ten low-income or mid-income countries receiving My Child Matters support: a descriptive study. Lancet Oncol.

[19] Ries LAG (1999). Cancer Incidence and Survival among Children and Adolescents: United States SEER Program 1975–1995.

[20] http://fondation-sanofi-espoir.com/en/ngo_child-matters.php.

[21] Steliarova-Foucher E (2005). International classification of childhood cancer. Cancer.

[22] Steliarova-Foucher E (2017). International incidence of childhood cancer, 2001-10: a population-based registry study. Lancet Oncol.

[23] Stiller CA, Childhood cancer and ethnic group in Britain: a United Kingdom Children’s Cancer Study Group (UKCCSG) study (1991). Br J Cancer.

[24] Stiller CA, Parkin DM (1990). International variations in the incidence of childhood lymphomas. Paediatric Perinatal Epidemiology.

[25] Stiller CA, Parkin DM (1992). International variations in the incidence of neuroblastoma. Int J Cancer.

[26] Newton R (2009). Geographical variation in the incidence of acute lymphoblastic leukaemia in childhood-Is it real?. Cancer Epidemiol.

[27] Wright DH (1967). The epidemiology of Burkitt’s tumour. Cancer Res.

[28] Cameron HM, Warwick GP (1977). Primary cancer of the liver in Kenyan children. Br J Cancer.

[29] Moore S (1997). Hepatocellular carcinoma in children. Ped Surg Int.

